# Supplementary material for: Maternal Milk Provision in the Neonatal Intensive Care Unit and Mother–Infant Emotional Connection for Preterm Infants
Source: Children (Basel). 2022 Feb 21;9(2):296. doi: 10.3390/children9020296 (PMC8870821; doi:10.3390/children9020296)
Supplement: Supplementary file 1 [file children-09-00296-s001.zip › Description S1 Factor Analysis.pdf]

S1. Supplemental Description of Factor Analysis

Correlations between the 25 items were small to moderate, with items correlated with at least 0.3 with at least one of item, and none were high (all correlations < 0.7) (Supplemental Table 1). The Kaiser-Meyer-Olkin measure of sample adequacy among the items was 0.8, and together with the results from the correlation matrix, suggested our data were suitable for structure detection using factor analysis.

To evaluate the dimensionality and underlying latent constructs of the Parenting in the NICU Study Survey among mother-infant dyads of preterm infants, we employed exploratory factor analysis using maximum likelihood as the estimation method and orthogonal (varimax, uncorrelated) rotation. The analytic sample included mother-infant dyads from the Parenting in the NICU Study born < 1500 grams and/or < 32 weeks' gestation with infant feeding data (n=127). A limitation of the analysis is our sample size (n=127) which is small to run such an analysis; adequate sample size guidelines suggest 10 times the number of variables being analyzed.[1] Due to communalities (h^2) > 1.0, we removed the item, "I don't find my baby cute," in order to proceed with the analysis. Among the preliminary eigenvalues, the first six had values greater than one and represented a cumulative variance of 100%. The values, in order, were 12.5, 5.2, 3.3, 1.6, 1.4, and 1,1, explaining 50%, 21%, 13%, 7%, 6%, and 4% of the variance, respectively. Assessment of the scree plot indicated a leveling off of the variance explained after four eigenvalues (Supplemental Figure 1). To ensure at least 3 items loaded onto each factor, we decided to retain four factors together having a cumulative variance of the preliminary eigenvalues equal to 91.1%. "The interpretation of rotated factor pattern as follows: 'maternal sensitivity' (Factor 1), 'emotional concern' (Factor 2), 'positive interaction/engagement' (Factor 3), and 'parenting detachment' (Factor 4) (Figure 2 and

Supplemental Table 2)". The (weighted) variance explained by each factor was 16.7, 6.0, 4.7, and 1.7, respectively. The study team collectively reviewed the four factors for conceptual clarity, item consistency, and together developed factor labels. The items loading onto the first three factors ('maternal sensitivity, 'emotional concern' and 'positive interaction/engagement') were conceptually clear, but 'parenting detachment' (i.e., Factor 4) appeared conceptually weak and confusing. In particular, the "not interested in child" item appears to be in the opposite direction from the other negatively framed items. As such, the study team excluded factor 4 and included factors 1-3 for primary analyses.

References

1. Floyd FJ, Widaman KF. Factor analysis in the development and refinement of clinical assessment instruments. Psychological assessment. **1995**, *7*, 286-299.
