# Supplementary material for: Maternal Milk Provision in the Neonatal Intensive Care Unit and Mother–Infant Emotional Connection for Preterm Infants
Source: Children (Basel). 2022 Feb 21;9(2):296. doi: 10.3390/children9020296 (PMC8870821; doi:10.3390/children9020296)
Supplement: Supplementary file 1 [file children-09-00296-s001.zip › Table S2 Parenting in the NICU Study Survey. Four latent variables and corresponding factor loadings.pdf]

Supplemental Table 2. Parenting in the NICU Study Survey. Four latent variables and corresponding factor loadings

Rotated Factor Pattern						
		**Factor1**	**Factor2**	**Factor3**	**Factor4**	**Final Communality Estimates (h^0)**
**nv_signl**	**Can tell from baby non-verbal signs when needs help/support**	**0.678**	-0.162	0.108	0.137	0.516
**bby_aln**	**Good at knowing when baby needs to be left alone**	**0.643**	-0.335	0.132	-0.073	0.549
**wthdrw**	**When baby crying/upset, I do not withdraw from relationship**	**0.235**	0.047	0.054	-0.110	0.072
**read_nd**	**Good at ready/interpreting baby's needs**	**0.944**	-0.119	0.055	-0.026	0.909
**unstld**	**Uncomfortable when baby unsettled/demanding**	-0.261	**0.350**	-0.189	0.342	0.343
**bby_cry**	**Can tell what is upsetting baby by his/her cry**	**0.513**	-0.333	0.024	0.154	0.398
**precious**	**Feel child is terribly precious**	0.062	**-0.265**	0.072	0.088	0.087
**tch_bby**	**Want to touch/hold baby when I see him/her**	-0.048	0.018	**0.843**	0.109	0.725
**care_fun**	**Taking care of baby is fun**	0.259	-0.005	**0.612**	-0.266	0.512
**do_nythg**	**Willing to do anything for child**	0.419	-0.181	**0.540**	-0.213	0.545
**miss_tch**[1]	**Miss touching/holding baby when not with me**	0.053	-0.185	**-0.765**	0.046	0.625
**peace**	**At peace when child nearby**	0.173	-0.236	**0.460**	-0.104	0.309
**enjy_int**	**Enjoy interacting w/baby**	0.128	-0.216	**0.395**	-0.104	0.230
**wrry_wys**[1]	**Worry about my child in many ways when child not w/me**	-0.019	**-0.208**	-0.204	-0.055	0.088
**nt_intsd**[1]	**Not that interested in child**	-0.107	-0.059	0.027	**-0.455**	0.223
**spk2bby**	**Speak to baby when caring fo0r him/her**	0.020	-0.059	-0.033	**0.119**	0.019
**ftr_anx**	**Thinking of future makes anxious about whether can be parent child needs**	-0.249	**0.532**	-0.039	0.150	0.369
**scrs_tch**	**Scares me to touch baby**	-0.075	0.169	-0.001	**0.596**	0.389
**dnk_int**	**Don't know how to interact w/ baby**	-0.339	0.422	-0.097	**0.582**	0.641
**trbl_own**	**Trouble actually feeling like this baby is mine**	-0.293	**0.411**	-0.271	0.327	0.436
**wrry_sk**	**Worry baby might get sick**	-0.027	**0.409**	0.158	0.093	0.202
**do4bby**	**Feel there is something more I should be doing for baby**	-0.089	**0.692**	-0.038	0.060	0.492
**dnk_2do**	**Sometimes don't know what to do for baby**	-0.163	**0.768**	-0.123	0.160	0.658
**hld_brk**	**Feel holding baby could break him/her**	-0.362	0.341	-0.054	**0.440**	0.443

[1.] Items 'I worry about my child in many ways when my child is not with me', 'I miss touching or holding my baby when s/he is not with me', 'I am not that interested in my child', and 'I don't find my baby cute' were reverse coded.
