# Supplementary figures and images for: Maternal Milk Provision in the Neonatal Intensive Care Unit and Mother–Infant Emotional Connection for Preterm Infants
Source: Children (Basel). 2022 Feb 21;9(2):296. doi: 10.3390/children9020296 (PMC8870821; doi:10.3390/children9020296)

Supplemental Figure 1. Scree Plot of Eigenvalues

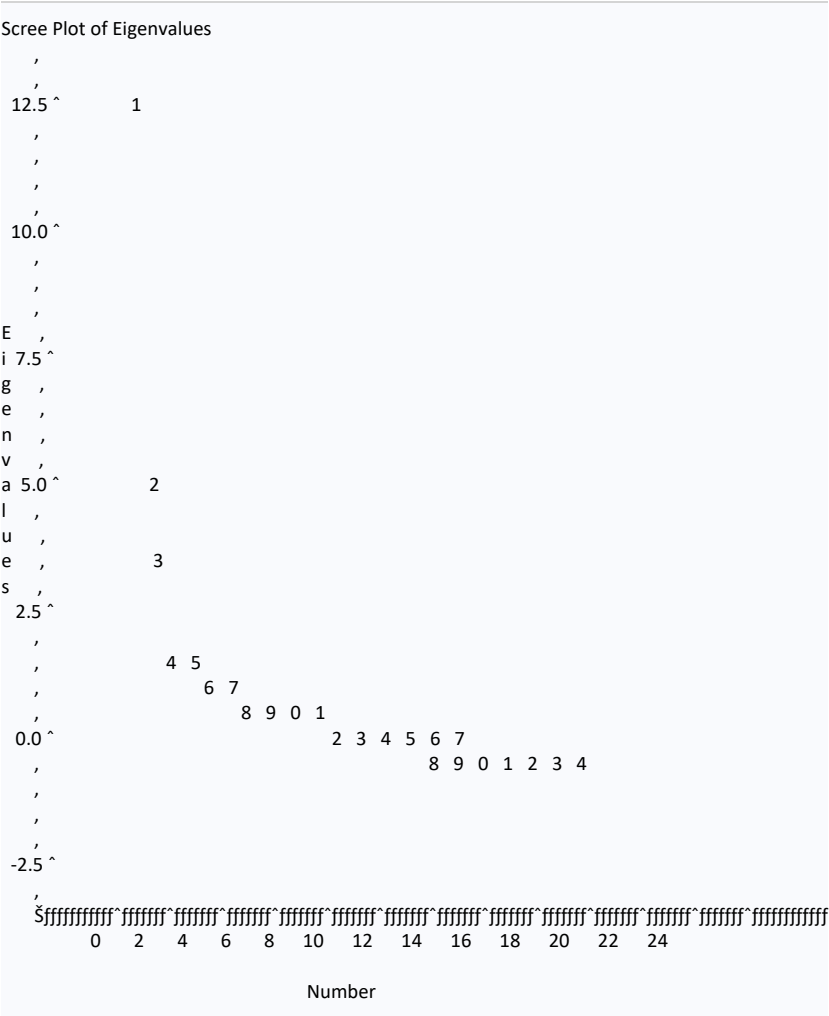

Supplement: Supplementary file 1 [file children-09-00296-s001.zip › Figure S1 Scree Plot of Eigenvalues.pdf]
